# Neuroprotective Effects of Carnosic Acid in an Experimental Model of Alzheimer’s Disease in Rats

**Published:** 2011-04-21

**Authors:** Nahid Azad, Homa Rasoolijazi, Mohammad Taghi Joghataie, Sara Soleimani

**Affiliations:** 1. Cellular and Molecular Research Center, Tehran University of Medical Sciences (Hemmat Pardis), Tehran, Iran; 2. Anatomy Department, School of Medicine, Tehran University of Medical Sciences (Hemmat Pardis), Tehran, Iran

**Keywords:** Carnosic Acid, ,, Alzheimer’s Disease, Hippocampus

## Abstract

**Objective:**

Alzheimer’s disease is the most common type of neurodegenerative disorder. It has been suggested that oxidative stress can be one of the pathological mechanisms of this disease. Carnosic acid (CA) is an effective antioxidant substance and recent studies have shown that its electrophilic compounds play a role in reversing oxidative stress. Thus we tried to find out whether CA administration protects hippocampal neurons, preventing neurodegeneration in rats.

**Materials and Methods:**

Animals were divided into four groups: Sham-operated (sham), CA-pretreated sham-operated (sham+CA), untreated lesion (lesion) and CA-pretreated lesion (lesion+CA). Animals in all groups received vehicle or vehicle plus CA (CA: 10mg/ kg) intra-peritoneally one hour before surgery, again the same solution injected 3-4 hours after surgery (CA: 3 mg/kg) and repeated each afternoon for 12 days. A lesion was made by bilateral intra-hippocampal injection of 4 µl of beta amyloid protein (1.5 nmol/µl) or vehicle in each side. 14 days after surgery, the brains were extracted for histochemical studies. Data was expressed as mean ± SEM and analyzed using SPSS statistical software.

**Results:**

Results showed that pretreatment with carnosic acid can reduce cellular death in the cornu ammonis 1 (CA1) region of the hippocampus in the lesion+CA group, as compared with the lesion group.

**Conclusion:**

Carnosic acid may be useful in protecting against beta amyloid-induced neurodegeneration in the hippocampus.

## Introduction

Alzheimer’s disease (AD) is the most common type of neurodegenerative disorder and one of the causes for senile dementia (1). This is a progressive and irreversible disease. The average life span after showing symptoms ranges between 1 and 25 years. Only a few drugs are available for Alzheimer’s disease treatment. They do not slow the progress of AD but are aimed at improving and stabilizing the memory and cognitive state of the patient by helping to retain and utilize the neurotransmitter acetylcholine (2). The aggregation and accumulation of the β amyloid (Aβ) protein has been implicated as the key pathogenic ‘trigger’ in this disease (3). The neuronal loss belongs not only to the diagnostic criteria, but has also been considered an important pathological component that should be replicated in an acceptable model of AD (4).

The Aβ cascade hypothesis was developed in the early 1980s. It suggests that senile plaques which are commonly observed in neurodegenerative abnormalities in AD can be developed following the accumulation of the Aβ peptide in the brain. This accumulation of Aβ is likely a consequence of an imbalance between production of Aβ from amyloid precursor protein (APP) and its removal process (5). Aβ induces the production of hydrogen peroxide and lipid peroxide in neurons and also the production of superoxide and proinflammatory cytokines in astrocytes as well as in microglia (6). The brain is the organ most susceptible to oxidative damage due to its high oxygen demand. Elevated oxygen consumption may lead to oxidative stress. Oxidative stress arises from an imbalance between cellular reactive oxygen species (ROS) production and the ability of cells to protect against this stress (7). Bilateral hippocampal damage demonstrated a significant decrease in the formation of new memories (8). Also, the subiculum and cornu ammonis 1 (CA1) region of the hippocampus are the regions that are most vulnerable to damage through AD (9).

Antioxidants may theoretically be useful in preventing the exacerbation of tissue damage. The candidate of antioxidant must penetrate the blood brain barrier (BBB) in order to reach a critical therapeutic level within the central nervous system (CNS). It must be administered as early as possible before the irreversible neuronal loss is observed (10). Rosemary (*Rosmarinus officinalis*) leaf extract shows very strong antioxidant activity. Carnosic acid (CA) is the most abundant antioxidant substance found in the leaves of the rosemary plant and is the main compound responsible for its antioxidant activity (11). Its radical scavenging activity follows a mechanism which is explained by the presence of two O-phenolic hydroxyl groups found at atoms C11 and C12, similar to the mechanism of other antioxidants (12).

Also, carnosic acid, as an electrophilic compound, could induce a Kip1\Nrf2 transcriptional pathway that takes part in the mechanism of antioxidant activity. This type of neuroprotection may have potential benefits in chronic neurodegenerative diseases (13). Therefore, the aim of the present study was to evaluate the beneficial effect of carnosic acid on neurodegeneration in the CA1 region of the hippocampus in an experimental model of AD in rats.

## Materials and Methods

### Materials

Aβ-protein fragment (1-40) and Cresyl violet acetate were purchased from Sigma Chemical Co. (Saint Louis, Missouri USA). Carnosic acid was purchased from A.G., Scientific Co. (San Diego, California, USA). Rabbit monoclonal caspase-3 antibody and rabbit IgG secondary antibody were purchased from Abcam Company (Cambridge, UK). Aβ (1-40) was dissolved in deionized water at a concentration of 1.5 nmol/µl, then divided into aliquots and stored at -80℃ before use. CA was dissolved in dimethyl sulfoxide (DMSO: 100 mg/ml) and stored at -20℃ before use. DMSO containing CA was diluted by phosphate buffered saline (PBS/DMSO: 10/1) immediately prior to injection.

### Experimental procedure

#### Animals

Male Wistar rats weighing 240-290 g were used (Pasteur Institute, Tehran, Iran) and were kept in the animal house of Tehran University of Medical Sciences (Hemmat Pardis). They were housed in the laboratory cages (3 animal/cage) under a 12 hours light/dark (LD) cycle, in room temperature of 21 ± 2℃ with free access to food and water.

Animals (n=20) were divided into four groups: Sham-operated (sham), CA-pretreated shamoperated (sham+CA), untreated lesion (lesion) and CA-pretreated lesion (lesion+CA). In this regard, animals in all groups received PBS+DMSO or PBS+DMSO plus CA (CA: 10mg/kg) intraperitoneally one hour before surgery, again the same solution injected 3-4 hours after surgery (CA: 3mg/kg) and repeated each afternoon for 12 days. For surgery, animals were anesthetized by injection of xylazine (20 mg/kg) and ketamine (100mg/kg) intra-peritoneally and positioned in a stereotaxic apparatus (Stoelting Co., USA). A bilateral lesion of the hippocampus was made by an injection of 4 µl of Aβ-protein fragment (1-40) or vehicle delivered by a 5 µl Hamilton syringe at the level of the hippocampus, for each side at the stereotaxic coordinates used in the Paxinos and Watson atlas (1986) : antero-posterior, -3.8 mm; lateral, ± 2.6 mm from bregma and -2.8 mm ventral from Dura with the incisor bar set at -3.3 mm. Each injection was made during a 5 minutes period and then kept in place for 5 minutes before being slowly withdrawn.

#### Histology

14 days after stereotaxic surgery, all rats were perfused through the ascending aorta with 4% Paraformaldehide in 0.1 M phosphate buffer (PB). Then the brains were extracted for histological studies and post-fixed in same solution. The paraffin slides were mounted onto gelatin-coated slides and stained with Nissle staining and caspase-3 antibody.

#### Nissle staining

We studied the coronal sections of the brain with 10-µm thickness, which were Nissle-stained with 0.1% Cresyl violet acetate. At least two sections representative of each two Paxinos and Watson atlas (1986) planes (-3.3 and -3.8; Bregma) were examined by scanning the entire extent on each side. Counting was done blind to the treatments received. The number of pyramidal cells in the CA1 region of the hippocampus was expressed as the total count obtained from the representative sections.

All sections were visually inspected using a light microscope (Olympus) with magnification of ×400 (in the area of 133530 µm²) using OLYSIA Bio Report Soft Imaging System GmbH, Version: 3.2 (Build 670).

#### Caspase-3 immunohistochemistry

The slides of one animal in each group were randomly selected and stained to show the caspase-3 positive neurons in the CA1 region. The protocol of Abcam Company was used. Briefly, after deparaffinizing and rehydrating the slides, a heat-induced epitope retrieval method was performed with sodium citrate buffer for 10 min at 98℃. Then the slides were washed in PBS+Triton X-100, blocked in normal serum + bovine serum albumin (BSA) + tris buffered saline (TBS), applied with primary antibody (rabbit monoclonal caspase-3 antibody) and incubated overnight. Subsequently, they were applied with rabbit IgG secondary antibody, counterstained with nuclear fast red, dehydrated, cleared and finally, mounted. Then all sections were observed using a light microscope (Olympus) with magnification ×400.

#### Ethical approval

All animal procedures were approved by the animal care committee of Chancellor for Research of Tehran University of medical science (Tehran, Iran).

#### Statistical analysis

Data were expressed as mean ± SEM and analyzed using SPSS statistical software (version 17). One-way analysis of variance (ANOVA) was used for comparison between all groups and post-hoc tests were carried out using Fisher’s least significant difference (LSD) test for each two groups. A difference of p<0.05 was regarded as significant.

## Results

All experimental animals tolerated surgical operation well with no mortality, due to the pre-treatments. Since the differences between results of mean numbers of CA1 neurons were not statistically significant for two sides (left and right) and also for two levels (-3.3 and -3.8), the mean of the results of the two sides and two levels are mentioned for each group.

**Fig 1:**
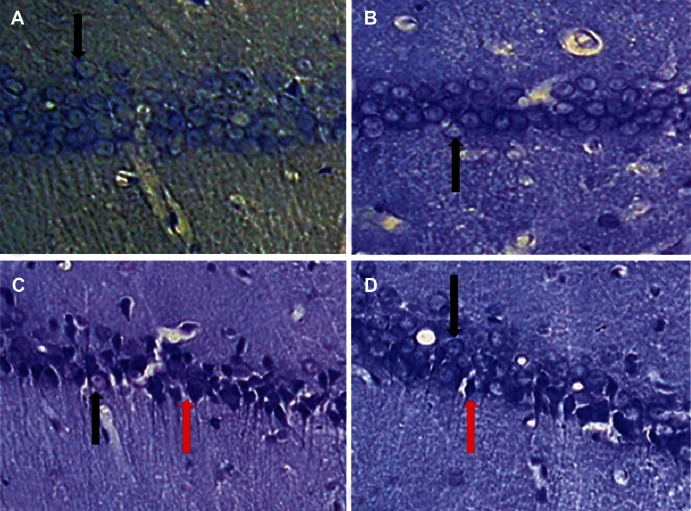
Photomicrographs of typical coronal sections through the CA1 area of the hippocampus showing Nissle-stained neurons in A: sham, B: sham+CA, C: lesion and D: lesion+CA groups. Black arrows show intact pyramidal cells and red arrows show degenerating pyramidal cells ×400.

**Fig 2:**
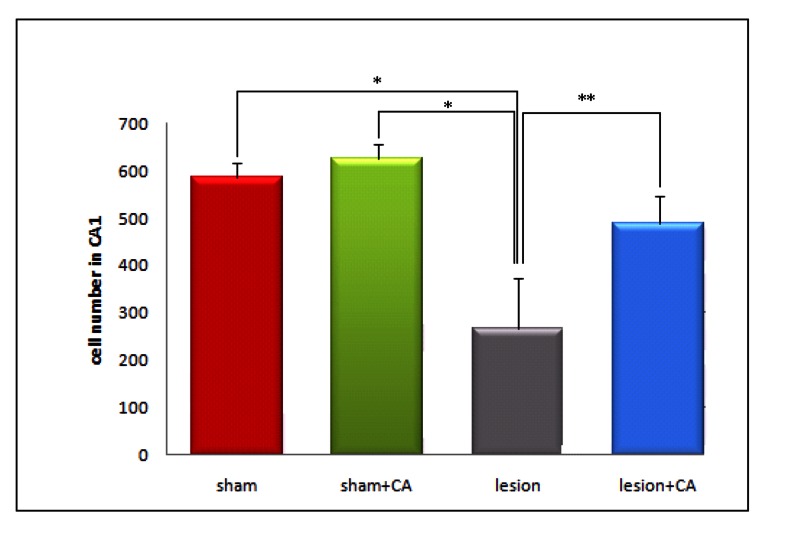
Means of pyramidal cell numbers in the CA1 region of the hippocampus on both sides (right and left) and at both levels (-3.3 and -3.8: Bregma) in studied groups (mean ± SEM). *p<0.01 and **p<0.05.

### Number of CA1 pyramidal cells

The results of neuronal counting showed the mean of total number of CA1 neurons for sham, sham+CA, lesion and lesion + CA groups were 587.3 ± 20.9, 627.4 ± 18.7, 268.2 ± 69.8, and 489.7 ± 33.2 respectively (Figs 1, 2). Thus, the mean of total number of cells showed a significant difference between all groups (p<0.01). There was a significant decrease in this parameter in the lesion group in comparison with sham and sham + CA groups (p<0.01). There was also a significant difference between lesion and lesion+CA groups (p<0.05). However, the differences between lesion+CA and both sham and sham + CA groups were not significant.

**Fig 3:**
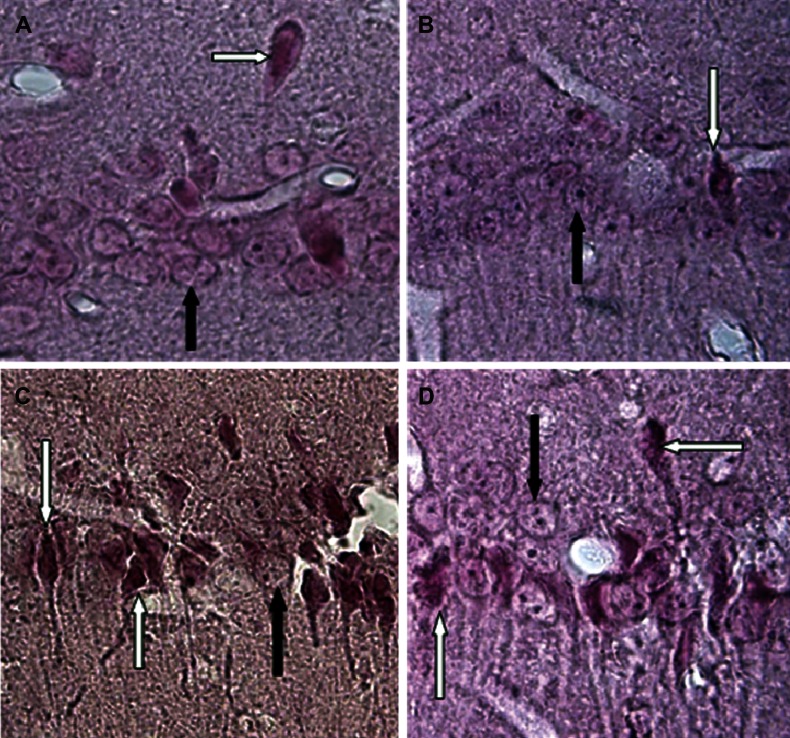
Photomicrographs of coronal sections of the CA1 region of the hippocampus (caspase-3 immunohistochemistry, counterstain with nuclear fast red). A: sham group; B: sham+CA group; C: lesion group; and D:lesion+CA group. Black arrows show intact pyramidal cells and white arrows show apoptotic pyramidal cells that express positive to caspase-3 antibody ×1000.

### Caspase-3 immunohistochemistry

The results of caspase-3 immunohistochemistry showed that there are comparatively many caspase- 3 positive neurons in the CA1 region in the hippocampus in the lesion group.

However, these caspase-3 positive neurons are reduced in the lesion+CA group. Also, there are few caspase-3 positive neurons in the sham and sham+CA groups (Fig 3).

## Discussion

The objective of the current investigation was to test the possibility that carnosic acid could have a protective effect in a model of AD. For this purpose, the number of Nissle-stained neurons of the CA1 region of the hippocampus was quantified. Also, caspase-3 immunohistochemistry was done to show cell death in the same region. There are two major conclusions to be drawn from the obtained results.

First, injection of 4µl of β-amyloid (1.5nmol/ µl) caused a significant reduction in Nissle-stained pyramidal neurons in the CA1 region as compared to sham and sham+CA groups. Also, caspase-3 immunostaining showed that cell loss in the CA1 region is partly due to the apoptosis induced by betaamyloid injection.

Previous studies have demonstrated that β-amyloid injection into the hippocampus led to neurodegeneration, damage and learning impairment (14). Aβ was shown to have a potential role in inducing oxidative stress and inflammation in the brain, which are present in the pathogenesis of Alzheimer's disease (6).

Secondly, neurons within the CA1 region of the hippocampus were largely protected against neurodegenerative effects induced by β-amyloid in the presence of carnosic acid. Thus, these results revealed that the considered hypothesis is true.

Recently, it has been reported that carnosic acid activates the Keap1/Nrf2 transcriptional pathway. Thus, CA can protect neurons from oxidative stress and excitotoxicity, both *in vitro* and in vivo. Furthermore, carnosic acid increases the level of reduced glutathione in the brain. Thus it is suggested to be a possible candidate for the treatment of neurodegenerative diseases with neuroprotective agents (13). Also, the quinone-type CA produced inside the cells is the most potent active form of CA that activates the Keap1/Nrf2 pathway (15). Furthermore carnosic acid (20 mg/kg/day, p.o.) reduced the body weight and accumulation of epididymal fat in high-fat diet-fed mice after 14 days (16). Also, it has been suggested that CA can be a powerful inhibitor of lipid peroxidation in microsomal and liposomal systems, as a scavenger of peroxyl radicals and H_2_O_2_. It also reacts with •OH in the deoxyribose system (17). It has been shown in an *in vitro* study that carnosic acid has direct action as an antioxidant (18) and also antiinflammatory potential on the level of gene regulation (19) and induced neural differentiation (20). Rosemary extract (carnosic acid and carnosol) can be considered to show low toxicity and no gross macroscopic lesions were observed at autopsy except fatty livers in mice subjected to repeated administration of rosemary extract (21).

## Conclusion

This study showed that bilateral injection of Aβ could induce neurodegenerative damage while intraperitoneal injection of carnosic acid could decrease neuronal death in the CA1 region of the hippocampus. These protective effects may be due to its antioxidant properties. Thus, carnosic acid might be used as a nutritional supplement.
